# Novel metal allergy patch test using metal nanoballs

**DOI:** 10.1186/s12951-014-0051-7

**Published:** 2014-12-03

**Authors:** Tomoko Sugiyama, Motohiro Uo, Takahiro Wada, Toshio Hongo, Daisuke Omagari, Kazuo Komiyama, Hitoshi Sasaki, Heishichiro Takahashi, Mikio Kusama, Yoshiyuki Mori

**Affiliations:** Department of Dentistry, Oral and Maxillofacial Surgery, Jichi Medical University, 3311-1 Yakushiji, Shimotsuke, Tochigi 329-0498 Japan; Advanced Biomaterials Department, Graduate School of Medical and Dental Sciences, Tokyo Medical and Dental University, 1-5-45 Yushima, Bunkyo-ku, Tokyo 113-8549 Japan; Department of Pathology, Nihon University School of Dentistry, 1-8-13 Kanda-Surugadai, Chiyoda-ku, Tokyo 131-8310 Japan; Nakayamagumi Co. Ltd., North 19, East 1, Higashi-ku, Sapporo, Hokkaido 065-8610 Japan; Graduate School of Engineering, Hokkaido University, North 13, West 8, Sapporo, Hokkaido 060-8628 Japan

**Keywords:** Nanoparticle, Nickel, Metal allergy, Patch test, Elemental distribution

## Abstract

**Background:**

Patch tests are often used in the clinical diagnosis of metal allergies. In currently available patch tests, high concentrations of metal salt solutions are used. However, diagnosis accuracy can be influenced not only by acute skin reactions to high concentrations of metal salt, but also by skin reactions to other components present in the patch or to pH changes. In this study, we developed Ni nanoparticles (termed “nanoballs”) for use in patch-test solutions.

**Findings:**

Highly soluble, spherical Ni nanoballs were prepared using plasma electrolysis. The Ni released from the nanoballs permeated through a dialysis membrane, and the nanoball-containing solution’s pH was maintained constant. Ni ions were released slowly at low concentrations in a time-dependent manner, which contrasted the rapid release observed in the case of a commercial patch test. Consequently, in the new test system, reactions caused by high concentrations of metal salts were avoided.

**Conclusions:**

By exploiting the high specific surface area of Ni nanoballs, we obtained an effective dissolution of Ni ions that triggered Ni allergy in the absence of direct contact between the nanoballs and mouse skin. This novel patch system can be applied to other metals and alloys for diagnosing various types of metal-induced contact dermatitis.

**Electronic supplementary material:**

The online version of this article (doi:10.1186/s12951-014-0051-7) contains supplementary material, which is available to authorized users.

## Background

**Figure 1:**
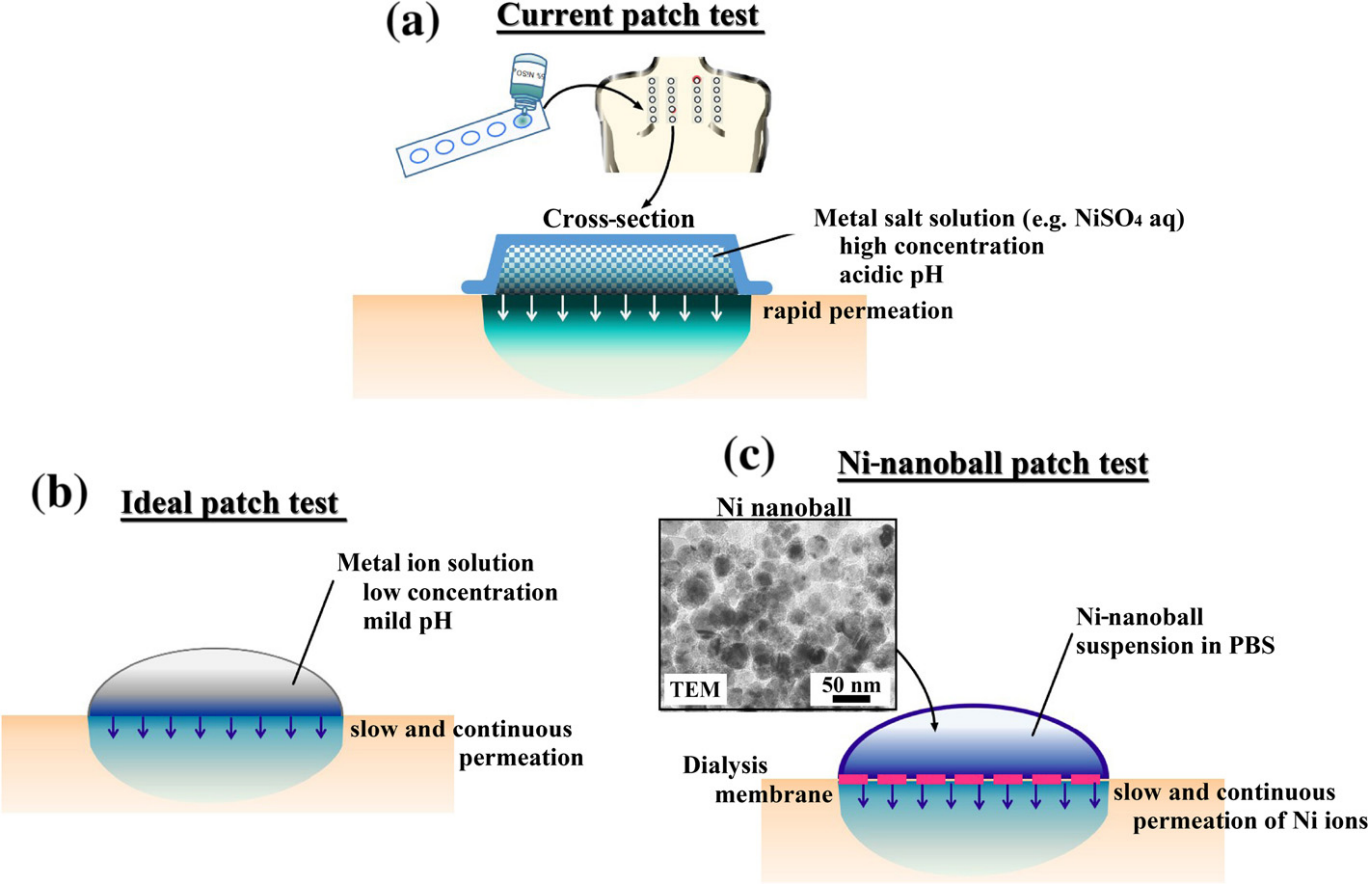
**Various patch**-**test schemes.** A currently available commercial patch test **(a)**, an ideal patch test **(b)**, and the novel patch test designed using Ni nanoballs **(c)**.

Metal-allergy patch tests are routinely used in the clinical diagnosis of metal-induced contact dermatitis. Currently available patch tests use high concentrations of various metal salts in aqueous solution. They contain a reservoir sheet that allows the test solution to permeate into the skin to induce a local allergic reaction (Figure 1a). A patch test solution typically contains a metal salt under an acidic condition [1]. Current patch tests can cause pustular or follicular reactions because of the high concentration of metal salts. In addition, false positive or negative reactions [2] and skin irritation often occur [3-5]. Metal allergies, however, are often triggered by metal ions that are continuously eroded from metallic materials under neutral pH conditions, and in this case, the metal ion concentration is typically low. Factors such as pH, metal-ion concentration, and dosage rates differ considerably between a genuine metal-allergic reaction and that which occurs in a patch test.

An ideal patch test must reproduce the metal erosion that occurs in metallic equipment (Figure 1b). Therefore, historically, large metal particles have been used as the metal source in the patch test [6]. However, their dissolution rates are inadequate for triggering an allergic reaction because of their low specific surface area [7,8].

To address the aforementioned limitations of patch tests, in this study, we prepared 40–50-nm-diameter Ni nanoballs (Figure 1c) through plasma electrolysis at the surface of electrodes [9]. Because of their size, we expected the Ni nanoballs to exhibit a high rate of Ni dissolution, which is suitable for a patch test. In our novel patch system, we used a Ni-nanoball suspension enclosed in a dialysis tube, which we call a “Ni-nanoball pack.” Ni ions released from the nanoball pack permeated through the dialysis membrane into the skin of mice, and thus direct contact between the Ni nanoballs and the skin was avoided. We evaluated this Ni-permeation behavior *in vitro* and also *in vivo* by using mouse skin. The Ni distribution in the skin was measured using synchrotron radiation-excited X-ray fluorescence (SR-XRF) analysis and particle-induced X-ray emission (PIXE), which are highly sensitive analysis methods. Furthermore, we examined the chemical state of the Ni nanoballs and the permeated Ni in the skin by performing X-ray absorption fine structure (XAFS) analysis.

## Methods

### Preparation of Ni-nanoball suspensions and an *in vitro* test of Ni-ion release

**Figure 2:**
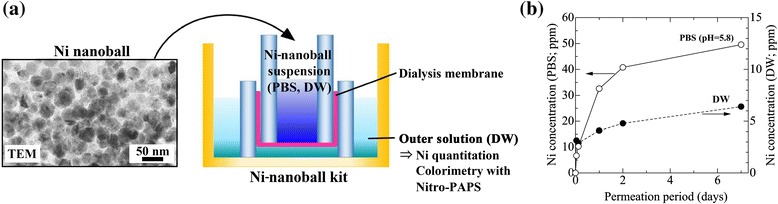
***In vitro*** **Ni release from Ni**-**nanoball suspensions through a dialysis membrane.** Experimental setup **(a)** and time-dependent release of Ni **(b)**.

Ni nanoballs were synthesized according to the method described by Toriyabe *et al*. [9] Ni nanoballs were dispersed in distilled water (DW) and 0.1 M phosphate buffer solution (PBS) at pH =5.8 to reach a concentration of 500 ppm of Ni nanoballs. In the *in vitro* test of Ni-ion release, the experimental setup used for examining Ni permeation through the dialysis membrane was prepared as shown in Figure 2a. The inner cylinder was filled with 500 μL of Ni-nanoball suspension in DW and PBS (pH =5.8) and immersed in 2500 μL of DW for 1 h to 7 days. Evaluation of the permeated Ni in the outer DW solution was based on a colorimetric reaction using 2-(5-Nitro-2-pyridylazo)-5-[N-n-propyl-N-(3-sulfopropyl)amino]phenol disodium salt dehydrate (Nitro-PAPS), which results in the Ni complex and shows a characteristic absorption around 568 nm (Additional file 1: Figure S1). Then, 500 μL of the outer solution was mixed with 2.0 mL of Nitro-PAPS aqueous solution (20 ppm), and the Ni concentration was estimated by the absorbance at 568 nm using the optical absorption (UV-Vis) spectrometer based on Yamashita *et al*. [10]. The standard Ni solutions (0.2-10 ppm) were prepared by diluting a 1 mg/mL Ni(NO_3_)_2_ solution with DW.

### *In vivo* Ni-permeation test designed for mouse skin

C57BL/6 mice (45–75 weeks old) were used in this study. All animal protocols were approved by the Animal Ethics Review Board of the Dental Hospital of Nihon University School of Dentistry, Tokyo, Japan, and conformed to the guidelines of the National Institutes of Health. The skin on the back of mice was depilated under general anesthesia. Approximately 20 mL of the Ni-nanoball suspension was placed into the dialysis tube and both ends were clamped. The Ni-nanoball pack was fixed onto the depilated back skin of the mice by using film dressing. The application period ranged from 30 min to 24 h, after which the mice were sacrificed. The Ni-nanoball pack was then removed and the skin was gently wiped, and the part of the skin that contacted the pack was excised and used for preparing frozen sections (20-μm thick) according to the method reported by Kubo *et al*. [11]. The sections were placed on Kapton film, dried and subjected to elemental-distribution analysis. As a control, a commercial patch test for Ni allergy (5% w/v NiSO_4_ aq.) was applied to mice using the same method. Adjacent specimen slices were stained with hematoxylin and eosin (H-E) and used for histopathological diagnosis. Detailed information on the chemicals, materials, and equipments used are presented in Additional file 1: Table S1.

### Elemental-distribution and chemical-state analyses

Elemental distribution analysis of the entire specimen was performed using SR-XRF. The specimen was irradiated with micro-focused X-ray and the specimen stage was scanned two- dimensionally. The fluorescence X-ray was detected at each point and the elemental distribution images were processed. The high-resolution elemental distribution was analyzed using micro-PIXE analysis, which involved exposure to the micro-focused proton beam with raster scanning over the target area of the specimen. The characteristic X-rays were detected, and the elemental distribution image was processed. The chemical states of the Ni contained in Ni nanoballs and the Ni in mouse skin were examined using XAFS analysis. Detailed conditions of the elemental analyses are shown in Additional file 1: Table S2.

## Results and discussion

The time-dependent release of Ni *in vitro* is shown in Figure 2b. We observed a continuous release of Ni. The Ni-dissolution rate of Ni nanoballs dispersed in PBS (pH 5.8) was higher than that of nanoballs dispersed in DW. This result agrees with the previous findings of previous studies showing that Ni solubility is increased in acidic solutions [12,13]. The pH of the commercial Ni patch-test solution was 3.8, which is lower than that of natural human skin, where the pH is typically below 5.0 [14]. Thus, we do not expect the Ni-nanoball suspension at pH 5.8 to irritate human skin. Furthermore, the Ni-release rate in our test system could be controlled by adjusting the pH.

**Figure 3:**
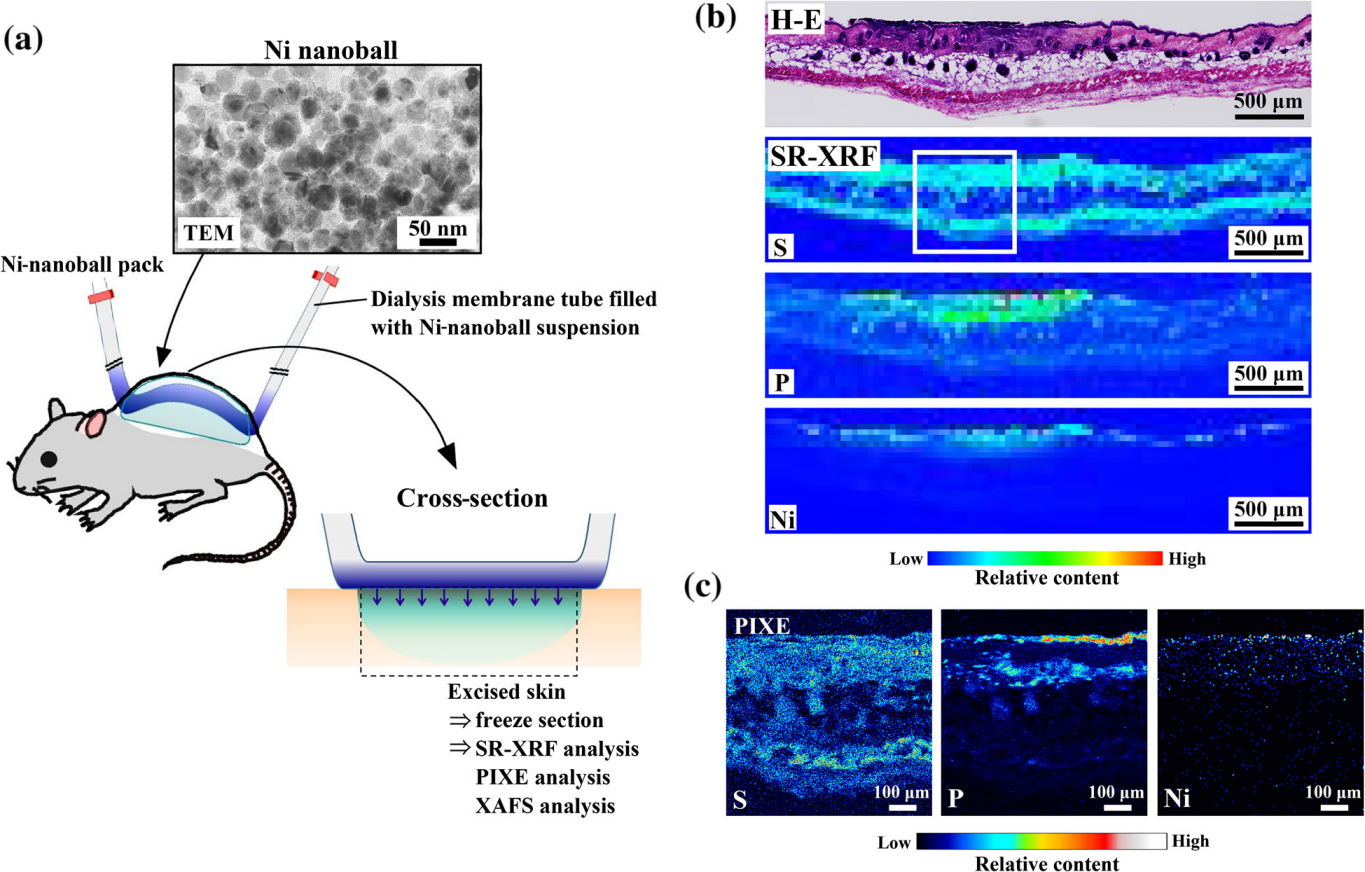
***In vivo*** **Ni permeation from Ni**-**nanoball packs into mouse skin after 24**-**h application.** Experimental setup **(a)**, histopathological (H-E) images and elemental-distribution images of skin cross-sections obtained using SR-XRF analysis **(b)**, and detailed elemental-distribution images obtained using micro-PIXE analysis **(c)**.

Figure 3b shows H-E-stained cross-sections of mouse skin (histopathological analysis) and their SR-XRF images of elemental distribution obtained after treatment with the Ni-nanoball pack for 24 h. The Ni that permeated from the patch was clearly observed on the surface side of the skin. Figure 3c shows the detailed elemental distribution of S, P, and Ni in the areas that exhibited high Ni accumulation (white squares in Figure 3b), as assessed using micro-PIXE analysis. The Ni concentration was high in the epidermis and spread into the dermis layer beyond the basal layer. These results indicate a clear internal permeation of Ni. Furthermore, the H-E-stained images of the same area showed a slight inflammatory response at the epidermis. The regions of inflammation overlapped with localized areas of Ni and P accumulation. Because the phosphate originates from inflammatory cells, this colocalization of Ni and P suggests that the Ni that permeates from the patch and penetrates the skin induces local inflammation.

**Figure 4:**
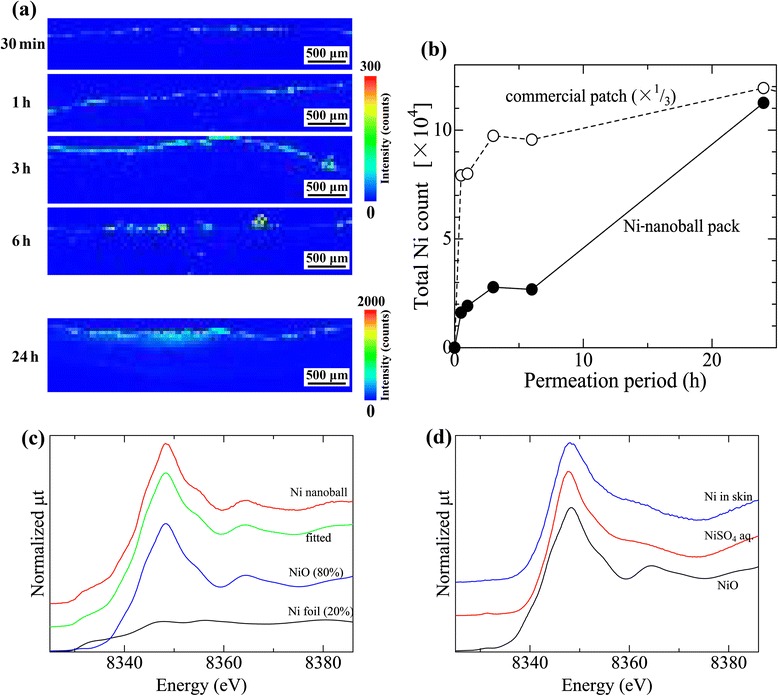
**Temporal change in the relative Ni content of the skin and the chemical states of Ni.** Time-dependent change in Ni distribution in mouse skin (cross-section) following 30-min to 24-h application of Ni-nanoball packs **(a)**; the time dependence of the total fluorescence X-ray intensity of Ni Kα (over the entire specimen area shown in Figure 4a) of the Ni-nanoball pack and a commercial patch **(b)**; and Ni K-edge XANES spectra of Ni nanoballs **(c)** and permeated Ni in mouse skin (released from a Ni-nanoball pack) **(d)**.

Figure 4 shows the temporal change in the relative Ni content of the skin. Figure 4a shows the images of Ni distribution in the skin cross-section, and Figure 4b shows the total fluorescence X-ray intensity of Ni Kα over the entire specimen area (shown in Figure 4a). When the Ni-nanoball pack was used, the Ni content in the skin increased linearly in a time-dependent manner until 24 h. Figure 4b shows the result obtained when we applied the commercial Ni-allergy patch to the skin by using the same method. The concentration of the Ni that permeated from the commercial patch increased drastically after application for only 30 min, and the permeated-Ni content was approximately three times higher than that after application for 24 h. This extremely high dose of Ni is likely to lower the accuracy of the commercial patch test as a result of the potential side reactions that it might cause. By contrast, the comparatively slow and time-dependent permeation of Ni from the Ni-nanoball pack is likely to be optimal for eliciting a Ni allergic reaction.

Figure 4 also shows the Ni K-edge XAFS spectra of Ni nanoballs (Figure 4c) and the permeated Ni in skin (Ni released from the nanoballs) (Figure 4d). The spectrum of the Ni nanoballs closely fits that of a mixture of metallic Ni (20%) and NiO (80%). This suggests that the Ni nanoballs were almost oxidized during storage. Conversely, the spectrum of the permeated Ni in skin (derived from the Ni-nanoball pack) was similar to that of NiSO_4_ aq., but distinct from that of NiO, a major component of Ni nanoballs. Therefore, the dissolved Ni ions were successfully released from the Ni nanoballs and they permeated the skin through the dialysis membrane.

During a genuine allergic reaction to metals, metal erosion and skin permeation occur in a low and continuous dose. We have shown that our Ni-nanoball pack releases Ni ions slowly from the suspension under mildly acidic conditions. Recently, the health risks posed by nanomaterials have become a growing concern [15-18]. Thus, in this study, we exploited the high specific surface area of Ni nanoballs in order to obtain an effective dissolution of Ni ions and used this to trigger Ni allergy without direct contact between the nanoballs and the skin.

## Conclusions

A high dose of metals and counter-ions and an acidic pH are factors that lower the accuracy of currently available metal-allergy patch tests. These factors were addressed effectively in this study, and the test system presented here could potentially serve as a state-of-the-art patch test. Another limitation of current patch tests is that they are available only for a few metal species, and the accuracy of these tests is still in question. The facile process used in this study for preparing Ni nanoballs can be applied to most pure metals and alloys, and then the method can be readily used for diagnosing other metal allergies. By conducting further studies on the ability to induce allergy in animal and human skin, the accuracy of the diagnosis obtained using our novel patch test designed for metal allergy could be potentially enhanced.

## Additional file

Additional file 1: Table S1 Detailed information on chemicals, materials, and equipment. **Table S2.** Detailed experimental conditions used for elemental analysis. **Figure S1.** Optical absorption spectra of Nitro-PAPS solution mixed with Ni ion solutions of various concentrations (a) and the calibration curve of Ni with the absorbance at 568 nm (b). Linear correlation was observed between 0 to 2.0 ppm of Ni. Quantitation of Ni was carried within this range by diluting the sample solution adequately.

## Abbreviations

SR-XRF: Synchrotron radiation-excited X-ray fluorescence
PIXE: Particle-induced X-ray emission
XAFS: X-ray absorption fine structure
PBS: Phosphate buffer solution
Nitro-PAPS: 2-(5-Nitro-2-pyridylazo)-5-[N-n-propyl-N-(3-sulfopropyl)-amino]phenol disodium salt dehydrate
