# Supplementary material for: Novel metal allergy patch test using metal nanoballs
Source: J Nanobiotechnology. 2014 Dec 3;12:51. doi: 10.1186/s12951-014-0051-7 (PMC4260209; doi:10.1186/s12951-014-0051-7)
Supplement: Additional file 1: Table S1 — Detailed information on chemicals, materials, and equipment. Table S2. Detailed experimental conditions used for elemental analysis. Figure S1. Optical absorption spectra of Nitro-PAPS solution mixed with Ni ion solutions of various concentrations (a) and the calibration curve of Ni with the absorbance at 568 nm (b). Linear correlation was observed between 0 to 2.0 ppm of Ni. Quantitation of Ni was carried within this range by diluting the sample solution adequately. [file 12951_2014_51_MOESM1_ESM.pdf]

Table S1 Detailed information on chemicals, materials, and equipment

Chemicals	Nitro-PAPS	Nitro-PAPS (Dojindo, Tokyo, Japan)
	Standard Ni solution	$Ni(NO_3)_2$ solution (1000 ppm, AAS grade, Kanto Chemical Co. Inc., Tokyo, Japan)
	Commercial patch test solution for Ni allergy	5% w/v $NiSO_4$ aq. (Torii Pharmaceutical, Tokyo, Japan)
Materials	Dialysis membrane (*in vitro*)	Cellu-Sep Flat Sheet Membrane (average pore size: 15–50 Å; MWCO: 12,000–14,000; thickness: 25 μm; Membrane Filtration Products, Inc., Texas, USA)
	Dialysis tube (*in vivo*)	Dialysis Membrane (average pore size: 25–50 Å; MWCO: 14,000; thickness: 20 μm; Wako Pure Chemical Industries, Ltd., Osaka, Japan)
	Film dressing	Opsite™ Post-Op Visible (Smith & Nephew, London, UK)
	Kapton film	12.5 μm (Du Pont-Toray Co., Ltd, Tokyo, Japan)
Equipment	Optical absorption spectrometer	56171-K, 57052-K, and 63723 (Edmond Optics, Barrington, USA)

Table S2 Detailed experimental conditions used for elemental analysis

SR-XRF analysis	Facility	BL-4A (Photon Factory, High Energy Accelerator Research Organization, Tsukuba, Japan)<br>Electron-storage ring operated at 2.5 GeV and 450 mA<br>Incident X-ray energy = 12.9 KeV<br>Focal size = 20 μm (obtained using polycapillary optics)<br>Measurement step = 40 μm (1-sec steps)
	Data processing	PyMca (ESRF, Ver. 4.7.3)
Micro-PIXE analysis	Facility	PASTA (National Institute of Radiological Sciences, Chiba, Japan)<br>Micro-focused proton beam<br>(3.0 MeV, 2-μm beam diameter, total irradiation = 200 nC)
	Data processing	OMDAQ2007 (Oxford Microbeams Ltd., Version 1.3.71.669)
XAFS analysis	Facility	BL-4A and BL-9A (Photon Factory, High Energy Accelerator Research Organization, Tsukuba, Japan)
	Methods	Ni nanoball, Ni foil, NiO (standard): Transmission method<br>Skin specimen, $NiSO_4$ aq. (standard):<br>Fluorescence method, a multi-element solid-state detector used (Canberra, Connecticut, USA)
	Standards	Ni (foil), NiO (reagent grade),<br>5% w/v $NiSO_4$ aq. (commercial patch test solution)
	Data processing	REX2000 (Rigaku, Ver. 2.5)

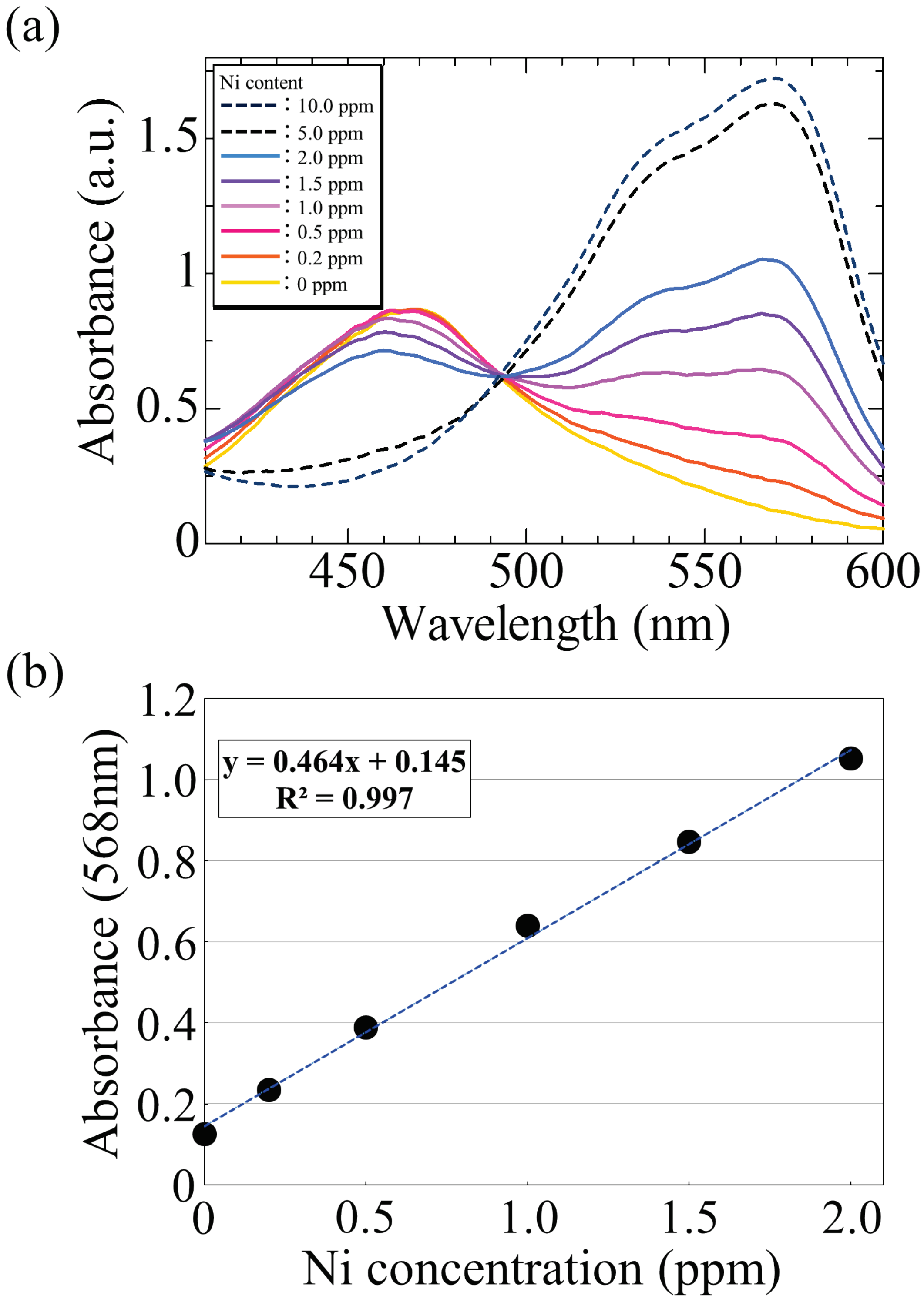

Figure S1 Optical absorption spectra of Nitro-PAPS solution mixed with Ni ion solutions of various concentrations (a) and the calibration curve of Ni with the absorbance at 568 nm (b). Linear correlation was observed between 0 to 2.0 ppm of Ni. Quantitation of Ni was carried within this range by diluting the sample solution adequately.
